# Systemic Bioenergetic Capacity Changes with Cognitive Status and Insulin Sensitivity in Older Adults

**DOI:** 10.1093/geroni/igab046.2423

**Published:** 2021-12-17

**Authors:** Gargi Mahapatra, Zhengrong Gao, James Bateman, Jenny L Gonzalez-Armenta, Allison Amick, Ramon Casanova, Suzanne Craft, Anthony J A Molina

**Affiliations:** 1 Sticht Center for Healthy Aging and Alzheimer's Prevention, Winston-Salem, North Carolina, United States; 2 Wake Forest School of Medicine, Winston-Salem, North Carolina, United States; 3 Wake Forest School of Medicine, Wake Forest School of Medicine, North Carolina, United States; 4 Wake Forest School of Medicine, Winston Salem, North Carolina, United States; 5 University of California, San Diego, La Jolla, California, United States

## Abstract

Systemic mitochondrial dysfunction is reported with AD progression, suggesting that peripheral blood cells may be used to investigate systemic mitochondrial alterations related to cognitive decline. We aimed to identify bioenergetic signatures associated with AD-related dementia and differences in insulin sensitivity associated with AD risk. We analyzed mitochondrial bioenergetics in peripheral blood cells collected from 365 older adults with varying cognitive status (normal, mild cognitive impairment, and AD) and insulin sensitivity. Normoglycemic individuals exhibited lower maximal bioenergetic capacity with AD (PBMCs: 239.6 pmol·min−1, p = 0.02; Platelets: 151.7 pmol·min−1, p = 0.06) compared to normal cognition (PBMCs: 271.5 pmol·min−1; Platelets: 171.7 pmol·min−1). Individuals with impaired insulin sensitivity exhibited lower maximal bioenergetic capacity in platelets with AD (171.6 pmol·min−1, p = 0.008) compared to normal cognition (210.6 pmol.min−1). Participants with impaired insulin sensitivity also exhibited unique bioenergetic profiles exemplified by overall higher levels of mitochondrial respiration, indicating that comorbidities such as diabetes can significantly influence bioenergetic capacity. We observed strong positive associations between maximal respiration in normoglycemic individuals with cognitive function, as measured by Modified Preclinical Alzheimer’s Cognitive Composite (mPACC5) (p = 0.06), and fatty acid oxidation in individuals with impaired insulin sensitivity with cortical thickness (p = 0.05). This study demonstrates that circulating cells may provide a cost-effective and minimally invasive way to monitor systemic bioenergetic changes associated with AD risk, progression, and insulin sensitivity. These findings also suggest that blood-based bioenergetics are related to key features of AD development and progression and should be further developed as a potential biomarker.

